# Supplementary figures and images for: Genomic, functional, and metabolic enhancements in multidrug-resistant Enterobacter bugandensis facilitating its persistence and succession in the International Space Station
Source: Microbiome. 2024 Mar 23;12:62. doi: 10.1186/s40168-024-01777-1 (PMC10960378; doi:10.1186/s40168-024-01777-1)

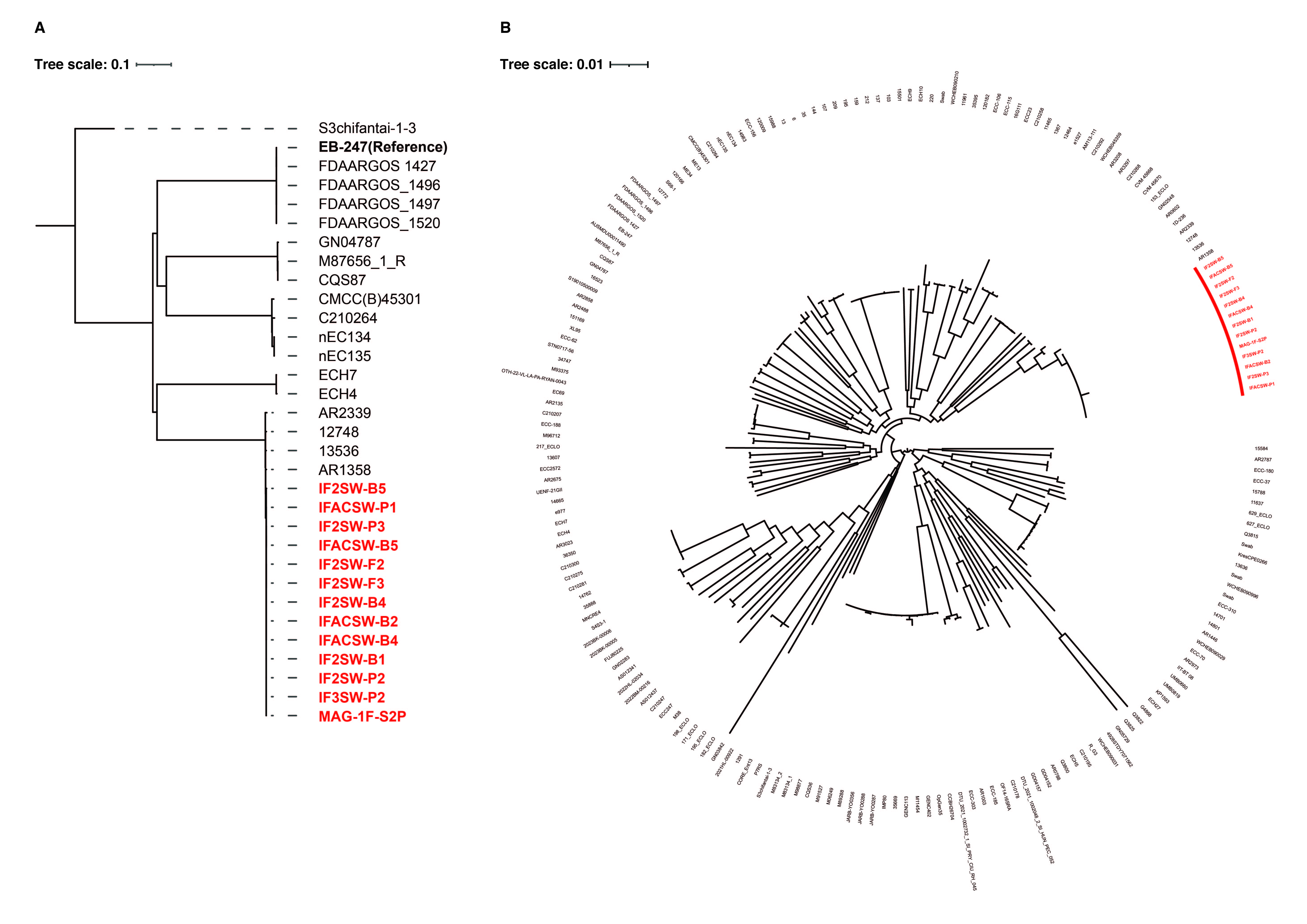

Supplement: Supplementary file 2 — Additional file 1: Supplementary Figure 1. Two alternate phylogenetic trees constructed from two different approaches. A. Phylogenetic tree constructed based on SNPs with selected genomes closely related to ISS E. bugandensis strains, with type strain EB-247T as the reference. B. Phylogenetic tree generated using the IQTREE algorithm from the multiple sequence alignment of core proteins derived from the pan-genome of E. bugandensis. In both cases, ISS strains are highlighted in red colour. [file 40168_2024_1777_MOESM1_ESM.jpg]

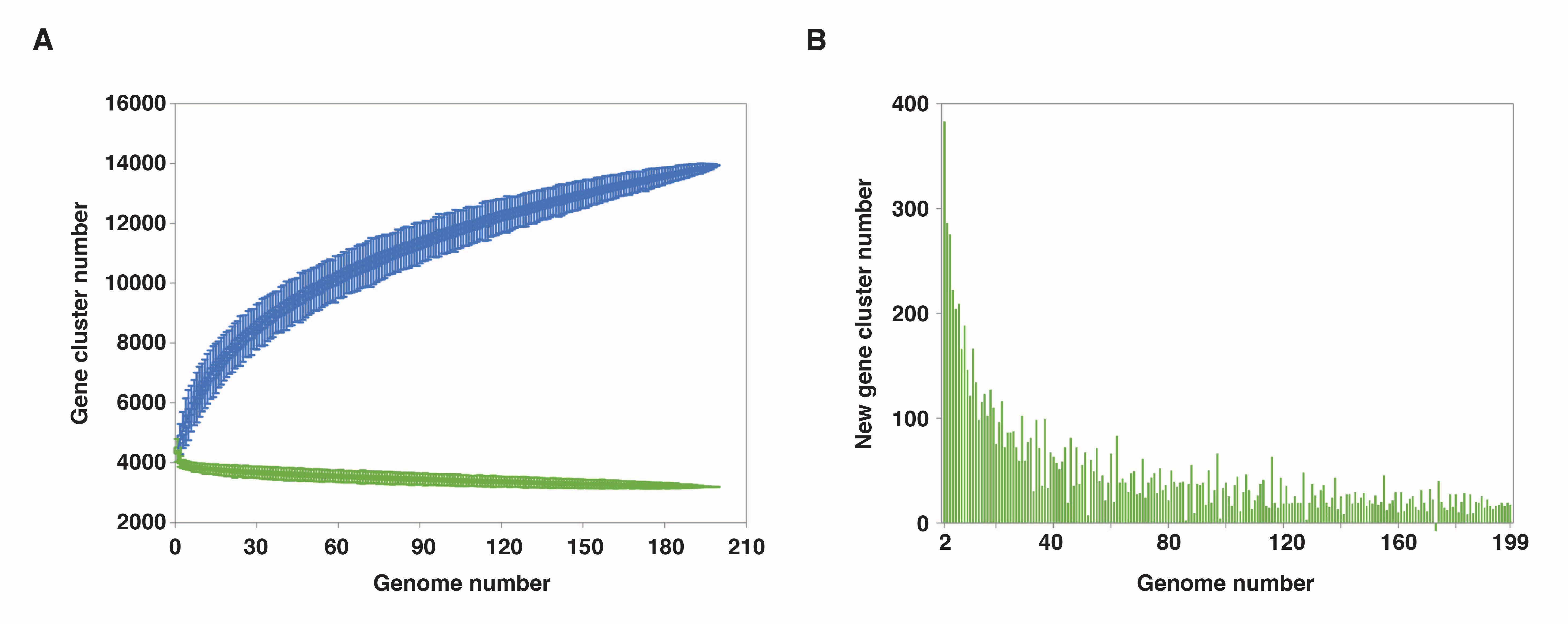

Supplement: Supplementary file 3 — Additional file 2: Supplementary Figure 2. Pan-genome analysis A. Core and accessory genomes of all E. bugandensis isolates. B. Number of new gene clusters introduced per genome. [file 40168_2024_1777_MOESM2_ESM.jpg]

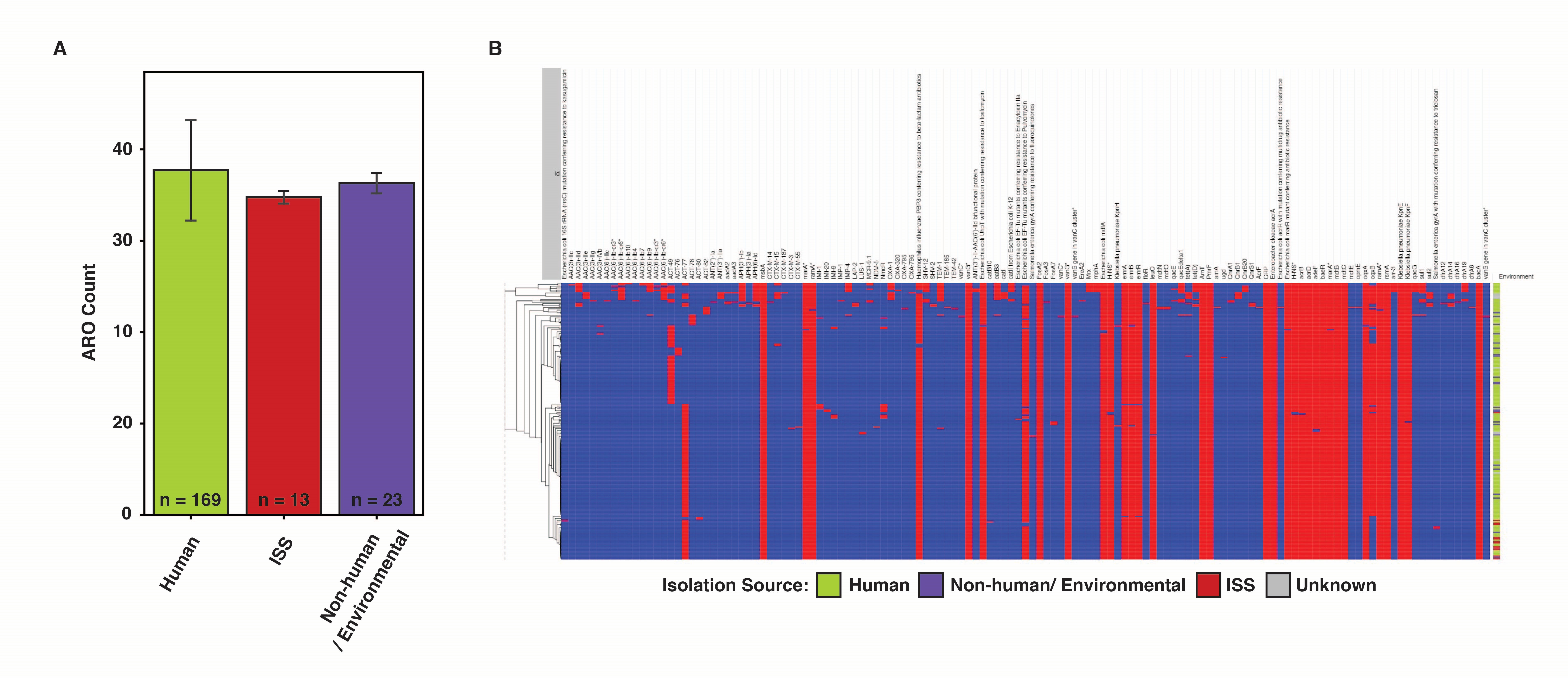

Supplement: Supplementary file 4 — Additional file 3: Supplementary Figure 3. Comparison between the number of AROs present A. ARO counts in ISS vs Non-ISS E. bugandensis. B. Presence/ absence of AROs across all 211 E. bugandensis genomes. [file 40168_2024_1777_MOESM3_ESM.jpg]

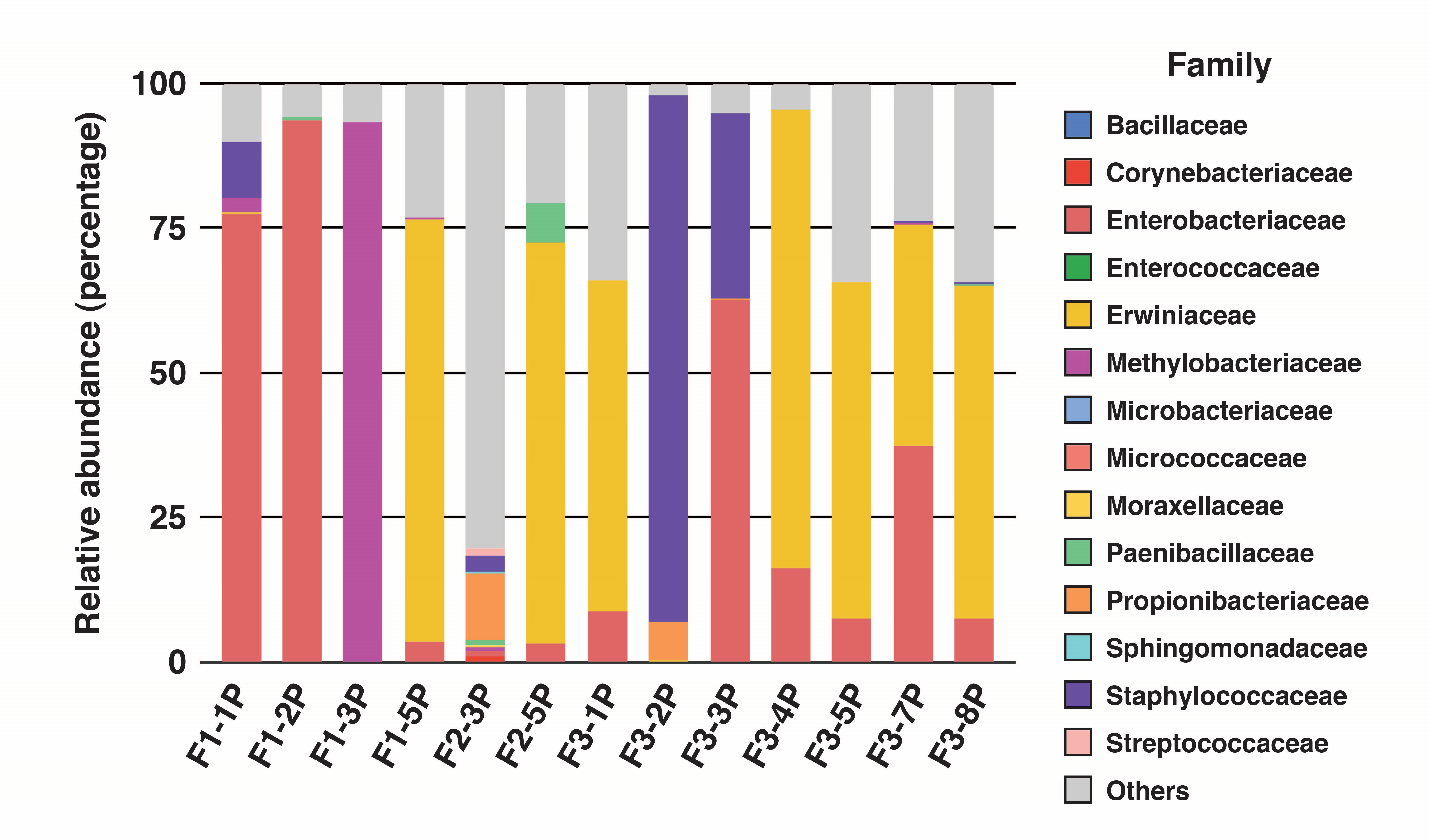

Supplement: Supplementary file 5 — Additional file 4: Supplementary Figure 4. The relative abundance of coexisting microbial families alongside E. bugandensis exhibits variations across distinct locations of ISS (with a confirmed presence of E. bugandensis). [file 40168_2024_1777_MOESM4_ESM.jpg]
